# Effects of donor breed and season of year on *in vitro* beef embryo production and recipient pregnancy rate

**DOI:** 10.1590/1984-3143-AR2025-0042

**Published:** 2026-04-10

**Authors:** Brenda Matos Fernandes, Gustavo Pereira Cadima, Natani Silva Reis, Ricarda Maria dos Santos

**Affiliations:** 1 Programa de Pós-graduação em Ciências Veterinárias, Universidade Federal de Uberlândia - UFU, Uberlândia, MG, Brasil; 2 Departamento de Medicina Veterinária, Faculdade de Medicina Veterinária e Zootecnia, Universidade Federal de Uberlândia - UFU, Uberlândia, MG, Brasil

**Keywords:** reproductive efficiency, embryo transfer, IVP

## Abstract

Approximately 85% of embryos produced in Brazil are *in vitro* (IVP). However, the success of IVP is still quite diverse, and several factors need to be coordinated to obtain a live calf from an oocyte. This study aims to evaluate the influence of the donor breed (Nellore *vs*. Senepol) and the seasons of year (Dry *vs*. Rainy) at the time of follicular aspiration (OPU) on the number of viable oocytes and embryos produced, and the recipients pregnancy rate per embryo transfer (P/ET). Data from 368 donors was evaluated, 198 Senepol and 170 Nellore, resulting in a total of 21,758 oocytes collected by OPU. During the period a total of 4,740 embryos were produced (2,135 Senepol and 2,605 Nellore) and 2,124 fresh embryo transfers were analyzed. A negative effect of dry season was observed on number of total and viable oocytes for Nellore. The embryo production rate was lower for Senepol in both periods. Nellore breed had lower embryo production rate in the rainy period. The P/TE was lower for embryos from Senepol donors during dry season, while the P/TE of embryos from Nellore donors was not affected by season. In conclusion, the dry season negatively influences total and viable oocyte collection in Nellore donors, while in Senepol donors remained constant throughout the year. The embryo production of Nellore donors is negatively influenced by rainy season, although it remains higher than the Senepol donors throughout the year, and P/TE of the embryos of Senepol donors is reduced during the dry season.

## Introduction

Based on IETS data, approximately 85% of embryos in Brazil came from *in vitro* production (IVP) in 2021. The number of IVP embryos has increased significantly in recent years, and North and South America accounted for 88.1% of all bovine embryos transfers (ET) in 2021. Among the trends for 2022, growth has been maintained (Viana, 2023).

IVP is a complex process which involves several stages and therefore is subject to challenges. Two important aspects of these challenges could be breeding issues and the seasonality effects on production (Mello et al., 2016). Moreover, several intrinsic factors in the technique may affect the results (Hansen, 2020). Although IVP, combined with other reproductive biotechniques, has contributed to intensifying production, the blastocyst production rate from oocytes has ranged between 30% and 50% over the years. Inconsistency in the oocyte quality has been identified as one of the main reasons for the limitation of IVP success. Therefore, further research is required to improve associated techniques to increase maturation and development capacity of recovered oocytes (Viana, 2018).

Donor breed could be a relevant factor in IVP success since different breeds can have different responses to assisted reproduction techniques. Variations in follicular development, oocyte quality and ovarian response can influence the results of IVP in different breeds (Hansen, 2020).

In addition to donor-related factors, environmental conditions have been consistently associated with variations in reproductive efficiency in bovine (Perea et al., 2026). Seasonal changes influence not only thermal stress and nutritional availability, but also endocrine patterns related to follicular development, oocyte competence and uterine receptivity (Copley et al., 2022). Studies evaluating conception rates after embryo transfer have demonstrated that environmental factors linked to seasonality may affect pregnancy outcomes, even when embryos are produced under controlled *in vitro* conditions (Copley et al., 2022; Zavaleta-Martínez et al., 2024).

Moreover, recent evidence suggests that cyclic environmental factors, such as lunar phases, may modulate reproductive responses, possibly through interactions with melatonin secretion, circadian rhythms and behavioral patterns (Perea et al., 2026). Although the biological mechanisms involved in these effects are not fully clarified, variations in conception rates according to season and lunar cycle have been reported in bovine embryo transfer programs, reinforcing the importance of considering environmental timing when evaluating reproductive outcomes (Zavaleta-Martínez et al., 2024; Perea et al., 2026). These findings highlight that reproductive efficiency results from a complex interaction between donor characteristics, embryo quality and environmental conditions at both oocyte recovery and embryo transfer.

According to the Brazilian Association of Meat Exporters in 2022, the Nellore breed, widely recognized in Brazil's agribusiness sector, currently leads in terms of the number of animals farmed nationally. Meanwhile, the Senepol breed, introduced to Brazil in the 2000s, has already gained a prominent position, ranking among the five most popular breeds for beef production (ABIEC, 2022). These breeds' adaptability to Brazil's tropical climate is one of the decisive factors behind Senepol and Nellore animals' popularity for beef production (Mello et al., 2016).

Considering the physiological and morphological characteristics of breeds that are suited for a tropical climate and the adaptability of Nellore animals over the years in Brazil's production system, we hypothesized that Nellore breeds' greater resistance to stress factors during follicular development would result in better quality oocytes and consequently more efficient *in vitro* embryo production, as well as higher pregnancies per embryo transfer when compared to Senepol breeds. Therefore, the aim of this study was to evaluate the effect of the donor breed (Nellore *vs*. Senepol) and the season of year (dry *vs*. rainy) at the time of follicular aspiration (OPU) on the number of viable oocytes and embryos produced, as well as the recipient pregnancy rate per embryo transfer (P/ET).

## Methods

The data analyzed in this study was provided by a commercial *in vitro* embryo production company located in Uberlandia, Minas Gerais, Brazil. Data was gathered by accessing Excel spreadsheets from the embryo production database held by the laboratory during 2021.

Data from 368 donors was evaluated, 198 Senepol and 170 Nellore, resulting in a total of 21,758 oocytes collected by OPU. During the period a total of 4,740 embryos were produced (2,135 Senepol and 2,605 Nellore) and 2,124 fresh embryo transfers were analyzed.

The aspirations were conducted at different times of the year and thus grouped into: Dry season (April to September) and Rainy season (January to March and October to December). The average annual temperature was 26 °C during the dry season and 19 °C to 20 °C during the rainy season, with rainfall concentrating in January and December with 309 mm and 292 mm respectively according to the Brazilian National Meteorological Institute – INMET (2021). The response variables considered were total and viable oocyte collected (TO and VO, respectively), viable oocyte rate (VOR), viable embryo production (VE), embryo production rate (EPR) and pregnancy by ET (P/ET) of the recipients.

The embryos were transferred fresh into recipients synchronized with an ovulation synchronization protocol based on progesterone and estradiol treatment. After synchronized ovulation, on day 7, the recipients were examined by transrectal palpation and ultrasound to determine the presence and size of the CL. Cows with CL larger than or equal to 15 mm were identified as potential recipients. Fresh embryos were transferred to the recipients, via transcervical by a deposition in the cranial portion of the uterine horn ipsilateral to the CL.

Pregnancy was diagnosed in the recipients by ultrasound examination 30 days after the date of synchronized ovulation. During the examination, the viability of the embryo was evaluated based on the heartbeat. Recipients diagnosed as pregnant were reexamined 60 days after the estrus.

The data was arranged according to the season of the year at the time of OPU: Dry season (01/04/2021 - 30/09/2021) and rainy season (01/01/2021 - 31/03/2021 and 01/10/2021 - 31/12/2021). It was considered the effect of each season (dry and rainy) and each donor breed (Nellore and Senepol) and the interaction between seasons and breeds on all the variables responses (TO, VO, VOR, VE, EPR, P/ET).

Total oocyte collected (TO), viable oocyte collected (VO), viable oocyte rate (VOR), viable embryo production (VE), and embryo production rate (EPR) were considered as continuous data, meanwhile pregnancy by embryo transfer (P/ET) was considered as binomial data. The continuous data that did not meet the assumptions of normality according to the Shapiro-Wilk test; therefore, it was submitted to the Kruskal-Wallis’s test and the binomial data was analyzed by the Chi-square test, both using the R software, including the effects of donor breed, season, and the interaction between breed and season in this model. Statistical differences were defined as *P* ≤ 0.05.

This study was approved by the Ethics Committee for the Use of Animals at the Federal University of Uberlandia (CEUA/UFU n° 23117.035192/2022-87).

## Results

Considering the total number of oocytes (TO), there was an effect of breed, an effect of season and an effect of the interaction between breed and season. The Senepol breed is not affected by the season, while the Nellore breed has a higher number of TO during the rainy season. Similar results were observed for the number of viable oocytes (VO), with the effects of breed, season and the interaction between them, where the Senepol breed is not affected by the season, and the Nellore breed has a higher number of VO during the rainy season (Table 1).

**Table 1:** Total oocytes number, viable oocytes number, viable oocyte rate, viable embryos number, embryo production rate and pregnancy rate per embryo transfer according to interaction of donor breed and season of the OPU.

**Donor Breed**	**Season**		**P-value**		
	**Rainy**	**Dry**	**Breed**	**Season**	**B vs. S**
	**Total Oocytes (n)**				
Nellore	34.67 ± 19.12	27.10 ± 14.90	S	S	S
Senepol	39.02 ± 21.57	40.22 ± 24.10			
	**Viable Oocytes (n)**				
Nellore	26.22 ± 15.59	18.83 ± 11.71	S	S	S
Senepol	28.64 ± 16.88	27.50 ± 16.38			
	**Viable Oocytes Rate (%)**				
Nellore	74.01	67.29	NS	S	NS
Senepol	71.81	67.82			
	**Viable Embryos (n)**				
Nellore	8.22 ± 6.83	7.36 ± 5.92	NS	NS	NS
Senepol	7.26 ± 6.55	8.44 ± 7.34			
	**Embryo Production Rate (%)**				
Nellore	31.94	37.74	S	S	S
Senepol	26.15	31.40			
	**Pregnancy per Embryo Transfer (%)**				
Nellore	37.90	35.20	NS	S	S
Senepol	42.83	29.15			

B = breed effect; S = season effect; B vs. S = breed and season interaction effect. S = significant; NS = not significant.

Regarding the viable oocyte rate (VOR), only the effect of the season was observed, with both breeds presenting a lower VOR in the dry season (Table 1).

The number of viable embryos (VE) was not affected by breed, season or the interaction between them. The embryo production rate (EPR) was affected by breed, season and the interaction between breed and season. The Nellore breed presented higher embryo production rate during the dry season, while the Senepol breed EPR remained the same during the dry and rainy seasons (Table 1).

Considering recipient pregnancy by embryo transfer (P/ET), it was observed that the pregnancy rate was not affected by the breed, although it was affected by the season and the interaction between breed and season. The Nellore breed was not influenced by the season, although the Senepol breed presented higher P/ET during the rainy season (Table 1).

## Discussion

This study evaluated the effect of donor breed (Nellore *vs*. Senepol) and season of year at the time of follicular aspiration (Rainy *vs*. Dry season) and their interaction on the number of total and viable oocytes collected, viable embryos produced, embryo production rate and pregnancy per embryo transfer of the recipients.

Regarding the collection of TO and VO, the Nellore breed produced more TO and VO during the rainy season. Studies show that taurine breeds have lower VO compared to zebu breeds (Batista et al., 2014, Sartori et al., 2016; Guimarães, et al., 2020), contrasting with this study, in which the Nellore breed had lower TO and VO in both seasons, and even lower during the dry season, compared to Senepol donors. Sales et al. (2015) states that *Bos indicus* females have a higher production of total and viable oocytes compared to *Bos taurus* females, as they undergo thermal stress during the warmest months of the year. Considering the results of this study, it might be possible that taurine animals are less affected by repeated OPUs, considering the amount of oocytes recovered from Senepol donors. Reis et al. (2006) comparing different OPU intervals with the number of oocytes collected from zebu and taurine cows, noted that the higher frequency of OPUs conducted in a short time interval in Nellore cows may have interfered with recovery time of the ovarian structures and the beginning of a new follicular wave, leading to a lower ratio of TO and VO when compared to taurine cows.

The differences between follicular dynamics, circulating progesterone concentration and LH pulsatility between zebu and taurine breeds should also be considered (Guimarães et al., 2020; Sartori et al., 2016; Batista et al., 2014), since these differences may interfere with follicular dominance timing and consequently on the quality of oocytes collected, depending on the stage of follicular wave in which OPU occurs.

Guimarães et al. (2020) recovered a larger quantity of high-quality oocytes per OPU from zebu donors, when compared to the quality and quantity of oocytes recovered from taurine donors (15.9 oocytes/donor *vs*. 11.4 oocytes/donor, respectively). These authors affirm that the variation in the quality of oocytes recovered between donor breeds may be related to the follicle population of each breed, as well as the amount of insulin produced and the amount of insulin-like growth factor type 1 (IGF-1), as well as the concentration of anti-mullerian hormone (AMH) produced by the granulosa cells, which controls the development and growth of follicles. Based this study's results, where Senepol breeds had higher TO and VO, these effects of the breed was observed probably because of the genetic composition Senepol donor, that is a *Bos Taurus* breed selected in a tropical environment in addition to the possibility of some thermotolerance genes being inherited from taurine breeds adapted to different climates, during the Senepol herds genetic selection. This targeted selection of superior animals has led to a consistent use of Senepol amongst livestock farms as presented on USDA research comparing rectal temperatures during grazing in summer months in Florida, where Senepol maintained their rectal temperature and feed intake behavior and even the crossbreed F1 heifers (Senepol x Hereford) presented similar results (Hammond et al., 1996).

The season and the breed of the donor and the interaction effect had no influence on the number of viable embryos (VE) produced by IVP. Berling et al. (2022), observing the effects of heat stress on embryo production in high-producing dairy cows, affirmed that the quality of oocytes from *Bos taurus* donors was affected by high temperatures, which compromised embryo development *in vitro*, though this effect was not observed in Nellore and Senepol donors, probably because those breeds are selected for beef production in tropical regions.

Nellore donors, despite producing fewer TO and VO in the dry season, presented higher EPR in the dry season compared to the rainy season, resulting in a similar number of VE produced for the breed in both periods of the year. These findings are interesting because despite Nellore donors being tolerant to heat stress, the best embryo production rate was observed during the dry period, and this fact should be further studied.

Regarding the recipients P/ET, this study identified the effect of season and the interaction between breed and season, with higher P/ET during the rainy season, especially for embryos produced from oocytes collected from Senepol donors during the rainy season. These results could be explained probably because of the embryo recipient nutritional management, since most of than are kept on pasture throughout the year. The seasonal effect observed on P/ET in the present study agrees with previous reports demonstrating that environmental conditions at the time of embryo transfer play a crucial role in pregnancy establishment (Copley et al., 2022; Zavaleta-Martínez et al., 2024).

Studies evaluating conception rates in beef cattle have shown that seasonal variation can influence uterine environment, recipient physiology and embryo–maternal signaling, ultimately affecting pregnancy success (Perea et al., 2026). Similar results were presented by Watanabe et al. (2017), in a retrospective data analysis of Nellore and Senepol cows comparing IVP and recipients P/ET results, concluded that during the rainy season, the P/ET of Senepol embryos was higher compared to the P/ET of Nellore embryos. Therefore, the higher pregnancy rates observed during the rainy season, particularly for embryos from Senepol donors, may reflect a more favorable physiological and environmental condition for embryo implantation and maintenance, consistent with literature indicating better conception under balanced seasonal conditions.

## Conclusion

It can be concluded that the dry season has a negative influence on the number of total oocytes and viable oocytes collected from Nellore donor, while the number of total and viable oocytes from Senepol donors remain constant throughout the year. The rate of embryo production from Nellore donor oocytes is lower in the rainy season, although it remains higher than the rate of embryo production from Senepol donor oocytes throughout the year, while the recipient P/ET of Senepol embryos is reduced during the dry season.

## Data Availability

Research data is only available upon request.
